# Vaginal Microbiota Is Stable throughout the Estrous Cycle in Arabian Mares

**DOI:** 10.3390/ani10112020

**Published:** 2020-11-03

**Authors:** Marta Barba, Rebeca Martínez-Boví, Juan José Quereda, María Lorena Mocé, María Plaza-Dávila, Estrella Jiménez-Trigos, Ángel Gómez-Martín, Pedro González-Torres, Belén Carbonetto, Empar García-Roselló

**Affiliations:** 1 Research Group-Microbiological Agents Associated with Animal Reproduction (ProVaginBio), Department of Animal Production and Health, Veterinary Public Health and Food Science and Technology (PASAPTA), Faculty of Veterinary Medicine, Cardenal Herrera-CEU University, CEU Universities, 46115 Alfara del Patriarca, Spain; marta.barba@uchceu.es (M.B.); rebeca.martinez@uchceu.es (R.M.-B.); juan.quereda@uchceu.es (J.J.Q.); mmoce@uchceu.es (M.L.M.); maria.plaza@uchceu.es (M.P.-D.); estrella.jimenez@uchceu.es (E.J.-T.); angel.gomezmartin@uchceu.es (Á.G.-M.); 2 Microomics Systems S.L, 08003 Barcelona, Spain; pedro.gonzalez@microomics.eu (P.G.-T.); belen.carbonetto@microomics.eu (B.C.)

**Keywords:** horses, equine, microbiome, metagenomic, estrus, diestrus, reproductive, vagina

## Abstract

**Simple Summary:**

Knowing which bacteria dominate vaginal microbiota and its variation throughout the cycle is important to study how to prevent reproductive diseases. In women, vaginal microbiota is dominated by *Lactobacillus* but this does not happen in other animals. Little is known about equine vaginal microbiota. The aim of this study was to describe the dynamics of equine vaginal microbiota during the ovarian cycle. Eight healthy adult Arabian mares were used to characterize vaginal microbiota by standard microbiologic and metagenomic procedures. The abundance of *Lactobacillus* was < 2% by both methods, meaning that equine vaginal microbiota was not dominated by these bacteria. Dominant bacteria included other genera such as *Porphyromonas* and *Campylobacter* among others. No changes in vaginal microbiota composition were found, suggesting that equine vaginal microbiota was stable throughout the ovarian cycle.

**Abstract:**

Lactic acid bacteria (LAB) dominate human vaginal microbiota and inhibit pathogen proliferation. In other mammals, LAB do not dominate vaginal microbiota, however shifts of dominant microorganisms occur during ovarian cycle. The study objectives were to characterize equine vaginal microbiota in mares by culture-dependent and independent methods and to describe its variation in estrus and diestrus. Vaginal swabs from 8 healthy adult Arabian mares were obtained in estrus and diestrus. For culture-dependent processing, bacteria were isolated on Columbia blood agar (BA) and Man Rogosa Sharpe (MRS) agar. LAB comprised only 2% of total bacterial isolates and were not related to ovarian phases. For culture-independent processing, V3/V4 variable regions of the 16S ribosomal RNA gene were amplified and sequenced using Illumina Miseq. The diversity and composition of the vaginal microbiota did not change during the estrous cycle. Core equine vaginal microbiome consisted of Firmicutes, Bacteroidetes, Proteobacteria and Actinobacteria at the phylum level. At the genus level it was defined by *Porphyromonas, Campylobacter, Arcanobacterium, Corynebacterium, Streptococcus, Fusobacterium*, uncultured *Kiritimatiaellae* and *Akkermansia*. *Lactobacillus* comprised only 0.18% of the taxonomic composition in estrus and 0.37% in diestrus. No differences in the relative abundance of the most abundant phylum or genera were observed between estrus and diestrus samples.

## 1. Introduction

Endometritis is considered as one of the most frequent causes of subfertility in mares [1]. The three most important physical barriers of uterine defense are the vulva, the vaginal vestibule (caudal vagina) and the cervix. Although current reproductive protocols include vulvar washing with water and neutral soap, it is essential to pass through the vaginal vestibule, and manipulation of the cervix to carry out reproductive procedures. This would allow bacterial pathogens present in the vaginal tract to reach the uterus during artificial insemination or other manipulations [2]. Modulation of vaginal microbiota in human medicine has led to the prevention and treatment of vaginal and reproductive tract diseases [3].

Culture-dependent and culture-independent methods are complementary in describing vaginal microbiota in women, although culture-independent methods are more precise in detecting clinically relevant vaginal non-*Lactobacillus* species (e.g., *Atopobium vaginae, Sneathia sanguinegens* or *Prevotella* spp.) known to be associated with bacterial vaginosis in women [4]. Culture-based approaches can miss the great diversity present in both diseased and healthy reproductive tracts. On the other hand, culture-independent studies based on metagenomics have changed the understanding of the role of the microbiome in health, including the reproductive tract. Metagenomics have allowed one to study the link between dysbiosis (i.e., microbiota imbalance) and certain diseases [5]. Moreover, an increased comprehension of the reproductive microbiota may lead to novel antibiotic-free therapeutic approaches to improve reproduction procedures, such as the use of probiotics to promote healthier vaginal microbiota.

It is known that lactic acid bacteria (LAB) dominate human vaginal microbiota and inhibit opportunistic pathogen proliferation [6]. The estrogen peak in women is associated with a higher abundance of *Lactobacillus* spp. [7]. In other mammals, LAB do not dominate vaginal microbiota, however shifts of dominant microorganisms occur during the ovarian cycle [8,9,10,11]. Interestingly, the most abundant genera in the vaginal microbiota of ewes and cows were shown to be *Aggregatibacter* spp. and *Streptobacillus* spp. in one study [12] or *Ureaplasma* spp. and *Histophilus* spp. in another study [11], in contrast to human samples [13].

There is scarce information regarding equine vaginal or uterine microbiota. One culture-based approach study described the presence of LAB in vaginal equine samples such as *Lactobacillus* spp. and *Enterococcus* spp. [14], however the relative abundance of these bacteria in mares remains unknown. A recent study using metagenomics has revealed that a moderate diverse microbiome is present in equine uterine samples where no significant growth was obtained by aerobic culture [15]. Furthermore, the uterine microbiome composition was found to be very similar to populations found on the external cervical os, meaning that communication between the uterine lumen and cranial vagina during estrus may occur.

Further studies to describe the vaginal microbiome in healthy mares are needed before investigating how bacterial populations change in diseases such as endometritis or can be modulated for its treatment or prevention. The aim of this study was to describe autochthonous caudal vaginal microbiota in healthy mares. Our first objective was to characterize the vaginal microbiota by culture-dependent and culture-independent methods. Our second objective was to compare the vaginal microbiota in estrus and diestrus.

## 2. Materials and Methods

### 2.1. Animals and Experimental Design

Eight healthy adult Arabian cyclic mares aged 5–23 years old and weighing between 350 and 450 kg were studied during estrus and diestrus, between June and July 2018. Mares were kept in several paddocks with access to ad libitum water and fed alfalfa and grass hay. Three mares were maiden and five mares were multiparous. No history of previous reproductive or fertility problems was reported in any mare. The genital tract was examined transrectally using a B-mode ultrasound scanner (Sonosite NanoMaxx) with an 8 MHz linear array probe. All mares had ovulated at least once before the start of the study, which was confirmed by the presence of a corpus luteum (CL). Transrectal palpation, ultrasound examination and teasing were performed to determine estrus and diestrus, once a week (every 7 days), and each mare showed regular cycles. Each mare was sampled (vaginal samples and blood) twice, once in estrus and once in diestrus. Furthermore, plasma progesterone concentration was determined to confirm the ovarian phase. Diestrus was considered when progesterone concentration was >1 ng/mL, and estrus when <1 ng/mL [16]. Inclusion criteria for estrus consideration (follicular phase) included: follicle diameter ≥30 mm, endometrial edema (score of 1–3), positive teasing and progesterone concentration <1 ng/mL. Inclusion criteria for diestrus consideration (luteal phase) included detection of a CL, negative teasing and progesterone concentration >1 ng/mL. All mares had to fulfill all inclusion criteria for both cycle phases to be included in the study. Animal procedures were handled in accordance with the Spanish Department of Agriculture Guide for Care and Use of Animals in Research and were approved by the local animal welfare committee at the Universidad CEU Cardenal Herrera (ref: 2017/VSC/PEA/00245).

### 2.2. Progesterone Testing

Blood samples were taken from the jugular vein in heparinized tubes and were immediately placed on ice for transportation. Samples were centrifuged at 3500 rpm (2000× *g*) for 10 min and plasma was separated. Plasma samples were frozen at −80 °C until progesterone quantification using a by solid-phase, competitive chemiluminescent enzyme immunoassay (Immunlite^®^ 1000 Immunoassay System, Siemens Healthineers, Madrid, Spain). Mean and SD progesterone concentration in estrus was 0.30 ± 0.24 ng/mL and in diestrus, 4.27 ± 1.53 ng/mL.

### 2.3. Vaginal Sampling Procedures

Vaginal sampling was performed with animals restrained in the stocks. Contamination was prevented by vulvar cleaning with neutral soap and water before sampling and by avoiding contact with the vulva. Samples were obtained by gentle swabbing of the vaginal wall at the level of the vestibule (caudal vagina) for 30 s in sterile conditions, as previously described [11]. Three swabs were obtained for each sampling point (i.e., in estrus and in diestrus) and immediately placed in transport tubes. One swab was used for culture-independent processing, the second swab was used for culture-dependent bacterial isolation and the third swab was used for cytological analysis.

### 2.4. Cytological Analysis

For cytological examination, each cotton swab was moistened with 0.2 mL of 0.9% saline solution and gently rolled onto a clean glass microscope slide and air-fixed. The smears were fixed with methanol for hematoxylin and eosin staining (Tinción Rápida Grifols, Diagnostic Grifols, S.A., Barcelona, Spain) and were examined by a photomicroscope (Leica DM2000). Ten microscopic fields from each sample were studied at ×400 magnification to identify and count the number of epithelial cells (superficial or basal cells) and inflammatory cells (neutrophil and macrophages) per microscopic field and at ×1000 magnification to identify and count bacteria as previously described [17,18,19].

### 2.5. Culture-Dependent Processing

Each vaginal swab was homogenized in 1 mL of brain heart infusion broth (Scharlab, Barcelona, Spain) and vortexed for 1 min at maximum speed to suspend attached bacteria. Then, decimal dilutions in phosphate-buffered saline (PBS) were plated in 13.5 cm diameter Petri dishes containing the following media: Man Rogosa and Sharpe (MRS) agar (Scharlab, Barcelona, Spain) for the selective growth of lactic acid bacteria (LAB) and Columbia blood agar (BA; Dismalab, Valdemorrillo, Madrid, Spain) as a general bacterial growth medium. Plates were incubated for 48 h at 37 °C microaerobically and aerobically, respectively. The number of colony forming units (CFU/mL) was counted.

### 2.6. Culture-Independent Processing

For culture-independent processing, the third swab was frozen at −80 °C until DNA extraction for high-throughput sequencing [4].

#### 2.6.1. Library Preparation and Sequencing

DNA extraction was done using the DNeasy PowerLyzer PowerSoil Kit (Qiagen, Hilden, Germany) following the manufacturer’s instructions. Agitation using Tissue lyser II (Qiagen, Hilden, Germany) at 30 Hz/s for 10 min at 4 °C was performed. Mock community DNA was included as a control of library preparation (Zymobiomics Microbial Community DNA, ZymoResearch, Irvine, CA, USA). The amplification was done using primers specific to the V3-V4 regions of the 16S rRNA DNA (V3-V4-Forward 5′-TCGTCGGCAGCGTCAGATGTGTATAAGAGACAGCCTACGGGNGGCWGCAG-3′,V3-V4-Reverse 5′GTCTCGTGGGCTCGGAGATGTGTATAAGAGACAGGACTACHVGGGTATCTAATCC-3′).

The amplification reaction was performed in a 10-μL final volume with a 0.2 μM primer concentration. The PCR program included: 3 min at 95 °C (initial denaturation) followed by 25 cycles: 30 s at 95 °C, 30 s at 55 °C, and 30 s at 72 °C and a final elongation step of 5 min at 72 °C. The purification of PCR products was done using AMPure XP beads (Beckman Coulter, Nyon, Switzerland) with a 0.9× ratio according to the manufacturer’s instructions. Final elution from the magnetic beads was done with 32 μL of Milli-Q water and 30 μL of the eluate were transferred to a fresh 96-well plate. The used primers contained overhangs, which allowed the addition of full-length Nextera barcoded adapters for Illumina MiSeq sequencing during a second PCR step (with only 8 cycles). S Sequencing ready libraries had ~450-bp insert sizes. Second PCR products were purified with the SequalPrep normalization kit (Invitrogen, ThermoFisher Scientific, Waltham, MA, USA), according to the manufacturer’s protocol. Libraries were eluted to a 20-μL final volume and pooled for sequencing. Quantification of the final pool was done with the qPCR using Kapa library quantification kit for Illumina Platforms (Kapa Biosystems, Sigma Aldrich, Saint Louis, MO, USA) on an ABI 7900HT real-time cycler (Applied Biosystems, ThermoFisher Scientific, Waltham, MA, USA). Sequencing was performed using Illumina MiSeq with 2 × 300 bp reads 15% of PhIX control libraries used to increase the diversity of the sequenced sample. Negative controls were added from DNA extraction steps, including one control for the sample collection buffer and one blank DNA extraction control using sterile water. Negative controls for both PCR amplification steps were also included. Control PCR products were visualized in 1.5% agarose gel electrophoresis stained with SYBR Safe (Applied Biosystems, ThermoFisher Scientific, Waltham, MA, USA). No visible bands were observed.

#### 2.6.2. Amplicon Sequences Processing and Analysis

In brief, raw demultiplexed forward and reverse reads were processed using the following methods and pipelines as implemented in QIIME2 version 2019.4 with default parameters unless stated [20]. DADA2 was used for quality filtering, denoising, pair-end merging and amplicon sequence variant calling (ASV, i.e., phylotypes or Operational Taxonomic Units, OTUs) using the qiime dada2 denoise-paired method [20]. Q20 was used as quality threshold to define read sizes for trimming before merging (parameters: --p-trunc-len-f and --p-trunc-len-r). Reads were truncated at the position when the 75th percentile Phred score felt below Q20: 288 bp for forward reads and 226 bp for reverse reads. After quality filtering steps, average sample size was 18,778 reads (min: 3547 reads, max: 46,269 reads). ASVs were aligned using the qiime alignment mafft method [21]. The alignment was used to create a tree and to calculate phylogenetic relations between ASVs using qiime2 phylogeny fasttree method [22]. ASV tables were subsampled without replacement in order to even sample sizes for diversity analysis using qiime diversity core-metrics-phylogenetic pipeline. The smallest sample size was chosen for subsampling [23]. Unweighted and weighted Unifrac distances were calculated to compare community structure. Alpha diversity metrics: observed OTUs (i.e., richness), Pielou‘s evenness index and Shannon index were calculated. Taxonomic assignment of ASVs was performed using a Bayesian classifier trained with Silva database (i.e., 99% OTUs database) using the qiime feature-classifier classify-sklearn method [24]. Since the swab samples could contain vaginal tissue cells, phylotypes were filtered to discard contaminant Eukariota DNA-derived amplicons using Blast against the mentioned database with a 90% identity cutoff.

### 2.7. Statistical Analysis

#### 2.7.1. Cytological and Culture-Dependent Data

Normality of the data was assessed based on examination of histograms plots and the Shapiro–Wilk test. Normally distributed variables (basal and superficial cells) were expressed as mean and standard deviation (SD) and non-normally distributed variables (BA and MRS counts) were expressed as the median and interquartile range (ICR). Differences between basal and superficial cells observed in cytological examination in estrus and diestrus were compared by a paired *t*-test. BA and MRS counts between estrus and diestrus were compared by the Wilcoxon-signed rank test. Values of *p* < 0.05 were considered significant. Statistical analyses were performed using SPSS^®^ 24.0 (IBM Corporation, New York, NY, USA).

#### 2.7.2. Amplicon Sequencing Data

Differential abundance of taxa was tested using the Kruskal–Wallis non-parametric test on the relative abundance of taxa (total sum scale) [25]. After Kruskal–Wallis, Conover’s test with the false discovery rate Benjamini–Hochberg correction was added for pairwise comparison. Alpha diversity comparisons were performed using the Kruskal–Wallis non-parametric test. Beta diversity distance matrices were used to calculate principal coordinates analysis (PCoA) and to make ordination plots using R software package version 3.6.0 (R Foundation for Statistical Computing, Vienna, Austria, http://www.R-project.org). The significance of grouping based on composition and structure of the microbial communities was tested using Permanova. Permdisp test was used to identify location vs. dispersion effects [26]. Significant threshold was set at 0.05. BiodiversityR version 2.11-1 (Nairobi, Kenya), PMCMR version 4.3, RVAideMemoire version 0.9-7 and vegan version 2.5-5 packages were used.

### 2.8. Core Microbiome Calculation

The core microbiome at the phylum level was defined as phyla that were present in at least 80% of mares and with a relative abundance over 1% [27]. The core microbiome at the genus level was defined as genera that were present in at least 85% of mares and with a relative abundance over 0.1% [27].

### 2.9. Nucleotide Sequences

The data discussed in this publication have been deposited in the NCBI Sequence Read Archive (http://www.ncbi.nlm.nih.gov/Traces/sra/) and are accessible through accession number PRJNA629105.

## 3. Results

### 3.1. Vaginal Microbiome Characterization by Culture-Processing

LAB represented only 2% (range 0–25.7%) of the total isolated CFUs. Six out of eight mares (75%) presented LAB counts in at least one of two samples. Higher BA counts were observed during estrus (Median 224500, ICR 247,200 CFU/mL) compared to diestrus (Median 16525, ICR 111,375 CFU/mL; *p* = 0.012). Changes in MRS CFU/mL were not related to the ovarian phases.

### 3.2. Vaginal Microbiome Characterization by Non-Culture Processing

A total of 1,648,507 pair-end reads were obtained. After quality filtering, trimming and denoising steps, 746,902 reads remained. Paired-end reads were merged and after chimera removal, 417,549 merged reads were used for phylotype calling with DADA2 [28]. Finally, 4399 phylotypes were detected. Singletones and doubletones were removed before diversity analysis.

#### 3.2.1. Alpha Diversity

Rarefaction curves showed that the achieved sequencing depth and subsampling size were enough to observe the complete diversity present in the microbial communities. A plateau was reached for every alpha diversity metric calculated (Supplementary Materials Figure S1). In addition, Good’s coverage index reached 1 for every sample. There were no significant differences in alpha diversity metrics between estrus and diestrus samples (Figure S2).

#### 3.2.2. Beta Diversity

No significant differences in microbial community composition and structure were observed for the calculated distances between estrus and diestrus samples (Figure S3).

#### 3.2.3. Taxonomic Composition of Caudal Vagina (Vestibule) in Mares in Estrus and Diestrus

The V3-V4 region of the 16S rRNA gene used in this study allowed the detection of both Bacterial and Archaeal communities. Bacteria and Archaea were detected in 100% (16/16) and 93.75% (15/16) of vaginal samples, respectively.

**Figure 1:**
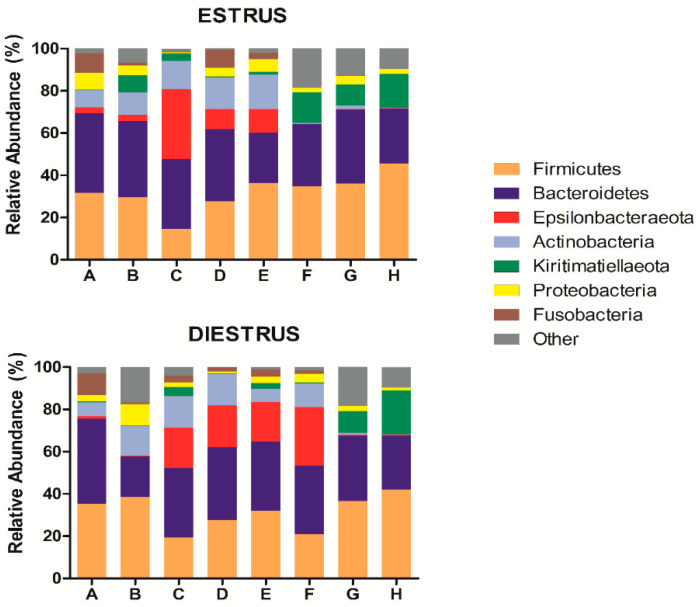
Phylum taxonomic relative abundance in individual mares in estrus and diestrus (only taxa with a mean relative abundance >1.5% at estrus or diestrus are represented). The letters in the x axis correspond to each individual mare.

**Figure 2:**
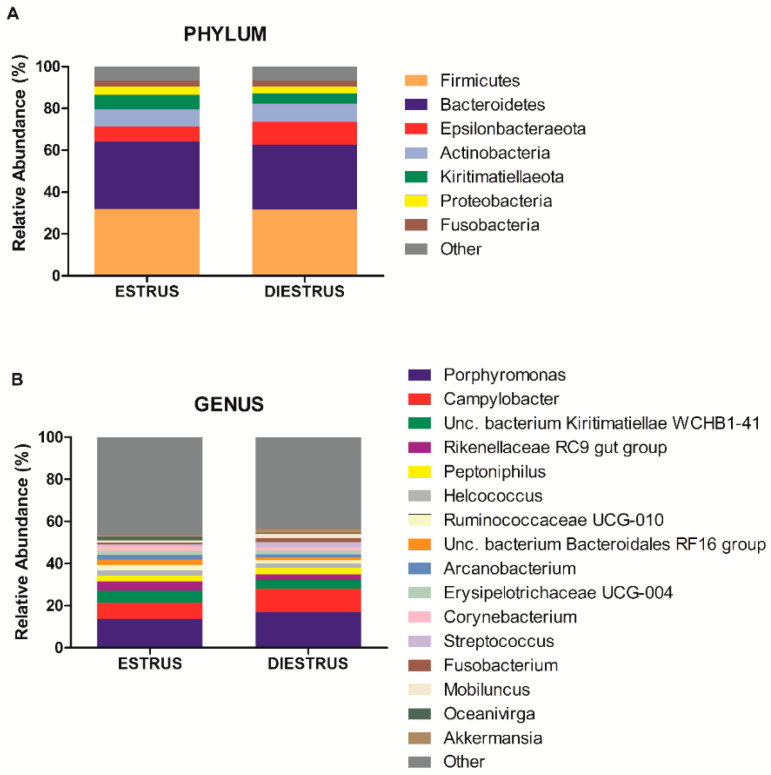
Mean bacterial phylum (**A**) and genus (**B**) taxonomic relative abundance classified by the ovarian phase. Only taxa with a mean relative abundance >1.5% at estrus or diestrus are represented.

**Figure 3:**
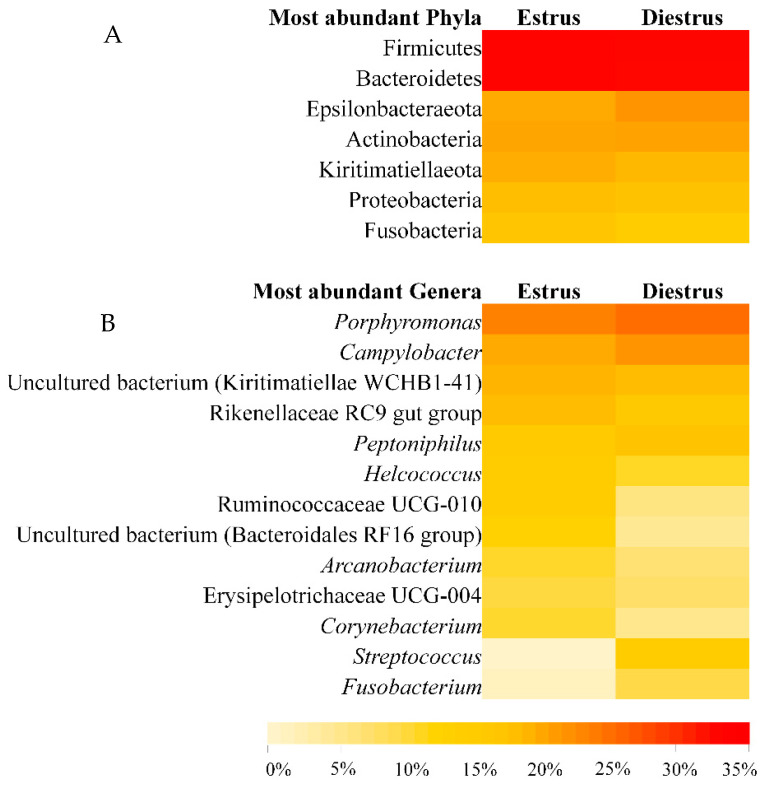
Heatmap of the mean relative abundance at the phylum level (**A**) and genus level (**B**) for equine vaginal samples at the different sampling points (estrus and diestrus). See color code scale based on the relative abundance on the bottom.

Twenty three bacterial phyla in vaginal samples in estrus (E) and 21 bacterial phyla in diestrus (D) were identified, from a total of 24 detected phyla. Firmicutes was the major phylum detected in all the animals in both phases (E: 32.03% mean relative abundance, D: 31.51%), followed by Bacterioidetes (E: 31.98%, D: 31.15%), Epsilonbacteraeota (E: 7.47%, D: 10.93%), Actinobacteria (E: 8.15%, D: 8.65%), Kiritimatiellaeota (E: 6.84%, D: 4.97%), Proteobacteria (E: 3.91%, D: 3.33%) and Fusobacteria (E: 2.87%, D: 2.70%; Figure 1). Remaining identified phyla comprised of <1.5% of the mean relative abundance at estrus and diestrus. As regards Archaea, the phylum Euryarchaeota was identified both in estrus (1.6%) and diestrus (1.45%). No differences in phylum composition were observed between estrus and diestrus samples (*p* > 0.05; Figure 2A and Figure 3A).

Bacteroidales was the most frequent order identified (E: 31.34%, D: 31.0%), followed by Clostridiales (E: 23.93%, D: 22.6%) and Campylobacterales (E: 7.47%, D: 10.93%). Lactobacillales represented only 2.79% of order taxonomic composition in estrus and 4.37% in diestrus. Three Archaea orders (Methanobacteriales, Methanomicrobiales and Methanomassiliicoccales) were identified both in estrus and diestrus, which represented less than 1% relative abundance.

**Figure 4:**
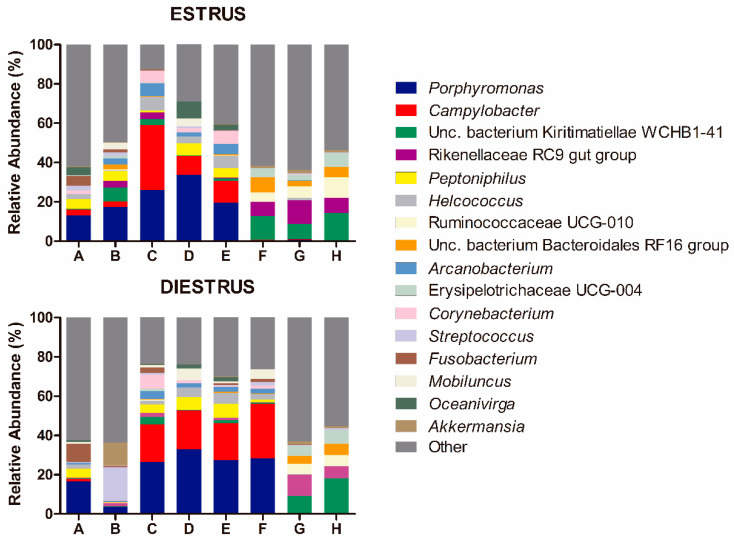
Genus taxonomic relative abundance in individual mares in estrus and diestrus (only taxa with a mean relative abundance >1.5% at estrus or diestrus are represented). The letters in the x axis correspond to each individual mare. Unc: uncultured bacterium.

**Figure 5:**
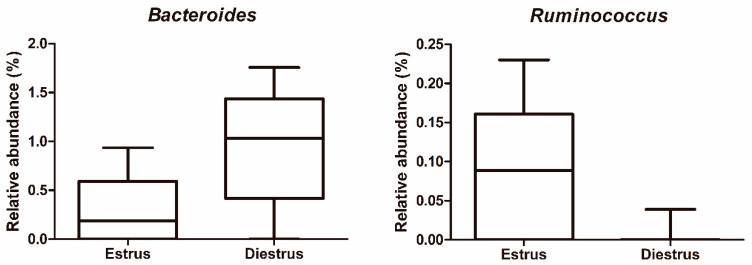
Differences in relative abundance of unidentified species of genera *Bacteroides* and *Ruminococcus* between estrus and diestrus in vaginal mare samples.

The most abundant genera in order of decreasing abundance were: *Porphyromonas* (E: 13.90%, D: 16.94%), *Campylobacter* (E: 7.46%, D: 10.93%), uncultured bacterium from *Kiritimatiaellae* WCHB1-41 (E: 5.77%, D: 4.22%), *Rikenellaceae* RC9 gut group (E: 4.33%, D: 2.75%), *Peptoniphilus* (E: 2.71%, D: 3.01%), *Helcococcus* (E: 2.61%, D: 2.16%), *Ruminococcaceae* UCG-010 (E: 2.68%, D: 1.50%), uncultured bacterium from Bacteriodales RF16 (E: 2.47%, D: 1.32%), *Arcanobacterium* (E: 2.14%, D: 1.63%), *Erysipelotrichaceae* UCG-004 (E: 2.01%, D: 1.72%), *Corynebacterium* (E: 2.11%, D: 1.40%), *Streptococcus* (E: 0.78%, D: 2.64%), *Fusobacterium* (E: 0.92%, D: 1.96%), *Mobiluncus* (E: 0.99%, D: 1.73%) *Oceanivirga* (E: 1.95%, D: 0.74%) and *Akkermansia* (E: 0.61%, D: 1.83%; Figure 4). Remaining genera comprised a mean relative abundance of <1.5% at estrus and diestrus. *Lactobacillus* represented only 0.18% of the genus taxonomic composition in estrus and 0.37% in diestrus. *Lactobacillus* was detected in 100% of animals in at least one time point (estrus or diestrus), six mares in estrus (75%) and six mares in diestrus (75%). However, relative abundance was low in all samples, with only one mare showing >1% relative abundance in diestrus and 50% of mares having <0.1%. At the genus level, no differences were observed between estrus and diestrus (*p* > 0.05) (Figure 2B and Figure 3B). At the species level, a decrease in relative abundance in estrus compared to diestrus was detected for an unidentified species of the *Bacteroides* genus (*p* = 0.024); in contrast, an increase in relative abundance in estrus compared to diestrus was detected for an unidentified species of the *Ruminococcus* 1 genus (*p* = 0.019; Figure 5).

Since no significant differences in the most abundant taxa between estrus and diestrus were identified, the mean relative abundance between estrus and diestrus of each mare was calculated to study the core vaginal microbiome. The core microbiome at the phylum level, consisted of Firmicutes (100%), Bacteroidetes (100%), Proteobacteria (100%) and Actinobacteria (87.5%). The core microbiome at the genus level consisted of *Porphyromonas* (87.5%), *Campylobacter* (100%), *Arcanobacterium* (87.5%), *Corynebacterium* (87.5%), *Streptococcus* (100%), *Fusobacterium* (87.5%), uncultured bacterium from *Kiritimatiaellae* WCHB1-41 (100%) and *Akkermansia* (87.5%).

It was observed that individually most animals kept the composition of the microbiome stable between both phases but two mares (B and F), which presented marked differences in taxonomic composition between estrus and diestrus (Figure 1 and Figure 4).

### 3.3. Vaginal Cytological Analysis

No animal showed cytological signs of inflammation based on low neutrophil and bacterial counts observed by microscopy. No differences in basal (E: 3.38 ± 1.87 cells/field, D: 2.86 ± 0.94 cells/field, *p* = 0.477) and superficial cells (E: 0.20 ± 0.19 cells/field, D: 0.19 ± 0.14 cells/field, *p* = 0.893) were observed in estrus compared to diestrus.

## 4. Discussion

The present study described for the first time, to these authors’ knowledge, individual fluctuations in equine vaginal microbiomes between estrus and diestrus. Furthermore, the vaginal microbiome was described in detail for healthy Arabian cycling mares, which had not been previously reported.

While the human vaginal microbiome has been well-described [29]⁠, the vaginal microbiome of few other species have been studied in depth using metagenomics approaches, including wild primates [30]⁠, mice [31], guinea pig [32], cow [11,33]⁠, bitch [34] and giant panda [35], but excluding most livestock. Firmicutes, Bacteroidetes and Proteobacteria usually are the predominant phyla reported in most studies. However, whereas *Lactobacillus* is the most abundant Firmicutes genus in women vaginal microbiota, this genus is not abundant in the other mammalian species [36]. This suggests that species-specific microbiota need to be studied deeper if we want to understand their influence in health and disease species-wide. There is scarce information regarding microbial composition in the equine reproductive tract. Most studies have focused on equine endometrial microbiome, showing that the uterine environment is not sterile. Moreover, some studies have compared uterine and vaginal samples but details were not published [5,15,37,38].

The data presented here is in agreement with previous studies of the equine reproductive tract microbiota. However, some differences are worth mentioning, even if this study did not aim to evaluate differences between uterine and vaginal microbiota, and comparisons between different studies using different methodologies are not advised. Core equine vaginal microbiome identified in this study consisted of Firmicutes, Bacteroidetes, Proteobacteria and Actinobacteria at the phylum level, which was in agreement with core equine uterine microbiome and postpartum vaginal microbiome previously reported [5,39]⁠. In the present study, core equine vaginal microbiome at the genus level was composed of *Porphyromonas, Campylobacter, Arcanobacterium, Corynebacterium, Streptococcus, Fusobacterium*, uncultured bacterium from *Kiritimatiaellae* WCHB1-41 and *Akkermansia.* Previously reported core endometrial microbiome in mares included *Pseudomonas, Porphyromonas* and *Streptococcus* [5]. While *Porphyromonas* and *Streptococcus* are shared between the vaginal core microbiome in this study and the previous reported uterine microbiome, *Pseudomonas* showed low relative abundance (<0.01%) in 100% of the individuals. Furthermore, the majority of the most abundant genera reported by Husso et al. 2020 in the vaginal microbiome of postpartum mares were also abundant in our study (*Corynebacterium, Porphyromonas, Helcococcus* and *Campylobacter)* [39]⁠. Factors such as geographic location and ethnicity have been reported to play an important role in the variation of composition and structure of the vaginal microbiota in women [40]. Further analysis should be done to define the influence of this type of factors in the vaginal microbiome.

The vaginal microbiome reported in this study show similarities with the previously described equine distal gastrointestinal tract fecal microbiome [41], which may lead to thinking that colonization of the vaginal tract occurs mainly by fecal contamination. Similar results have been found in a study carried out in pregnant mares, where the most abundant phyla (Firmicutes, Proteobacteria and Bacteroidetes) were shared between oral, vaginal, placental and fecal microbiomes [42]. Vaginal colonization by fecal bacteria was already proposed for other species [43]. In humans, gut microbiome dysbiosis has been associated with many diseases, including those in the reproductive tract [44]. Furthermore, it has been shown that gut microbiome dysbiosis is associated to increased blood estrogen concentration, which could alter reproductive function [45]. Moreover, fecal microbiota transplantation and oral probiotic administration has been suggested as a treatment for reproductive diseases in women [46]. Another hypothesis would be that the foal´s gut is colonized by bacteria from the mare´s vaginal tract during parturition, however a recent study demonstrated that the gut microbiome is probably influenced by several factors such as the dam´s fecal, oral- and vaginal microbiota in addition to other sources such as environmental [39]. Further studies are needed to describe these interactions in mares. Factors such as diet, which are known to affect the gut microbiome in horses, could also affect vaginal microbiome [47,48].

Similar to what has been observed for other mammalian species [9,11,12,30]⁠, *Lactobacillus* spp. did not dominate the equine vaginal microbiome. These results were consistent for both culture-dependent and culture-independent approaches used here. Despite the isolation of *Lactobacillus* spp. such as *L. pantheris, L. mucosae* and *L. equi* in vaginal equine samples has been previously reported [14]⁠, the relative abundance of these species in our samples was either low or null, which is in agreement with Husso et al. 2020 [39]. These results suggest that *Lactobacillus* spp. could have a restricted role in the mare´s vaginal tract and reinforce the idea that more efforts to detect other potential probiotic bacterial species in mares, and in other non-human mammalian species, are needed in order to explore new antibiotic-free therapeutic strategies.

Changes in diversity and composition of the vaginal microbiome due to fluctuations in estrogen levels have been widely described in women [49,50]. In other mammalian species, shifts of dominant microbiota occur during the ovarian cycle [8,9,10], as we recently described in Holstein cows [11]. However, studies carried out in minipigs showed that composition of the vaginal microbiota was very stable during an estrous cycle [9]. In our study, despite higher bacterial counts being observed in estrus with culture-dependent methods, no differences in microbiome composition were observed depending on ovarian cycle phases, which has never been reported before for mares. In mares, vaginal pH is very close to neutrality [14], while vaginal pH in women is low (median pH = 4.5), probably due to *Lactobacillus* spp. predominance in the vaginal microbiota [13]. Furthermore, vaginal pH decreases when there is high estrogen concentration (follicular phase and ovulation) in women [13]. In contrast, a decrease in vaginal pH was detected in one study the day of ovulation in mares, when estrogen concentration is low. However, no differences in pH in the rest of estrus period were observed compared to diestrus [51]. The lack of changes in pH in estrus compared to diestrus is in agreement with the lack of changes in microbial diversity and microbiome composition observed between these phases in our study. The ovarian cycle phase did not explain variation in vaginal microbiome composition and diversity, but other factors such as age, history of parturition and level of activity could be studied to investigate its possible effect in individual microbiome variation.

Our study supports the investigation of the vaginal microbiome in disease status since significant changes in the vaginal microbiome probably suggest dysbiosis related to disease or stress generated by reproductive procedures, rather than hormonal influence throughout the cycle, although this needs to be confirmed. Furthermore, the results of this study could be used to select potential probiotic bacteria that are shown to be part of dominant flora in a healthy status.

## 5. Conclusions

Our study has shown that the diversity and composition of the equine vaginal microbiota was stable throughout the estrous cycle. We also reported that LAB such as *Lactobacillus* spp. do not dominate equine vaginal microbiota, in contrast to what has been reported for humans but similar to other non-human mammalian species. Further investigation on the microorganisms presented here as dominant and frequent should be done to describe their potential role in health in the face of disease prevention strategies.

## Supplementary Materials

The following are available online at https://www.mdpi.com/2076-2615/10/11/2020/s1, Figure S1: Rarefication curves comparing richness of diestrus (red) and estrus samples (blue), Figure S2: Alpha diversity analysis. Observed OTUs (i.e., richness), Shannon index and Pielou‘s index (evenness) comparison between diestrus and estrus samples, Figure S3: Beta diversity analysis. Principal coordinate analysis based on unweighted Unifrac, weighted Unifrac, Bray Curtis and Jaccard distance matrices. Samples at estrus (red) and diestrus (blue) are compared.
