# Supplementary figures and images for: Vaginal Microbiota Is Stable throughout the Estrous Cycle in Arabian Mares
Source: Animals (Basel). 2020 Nov 3;10(11):2020. doi: 10.3390/ani10112020 (PMC7692283; doi:10.3390/ani10112020)

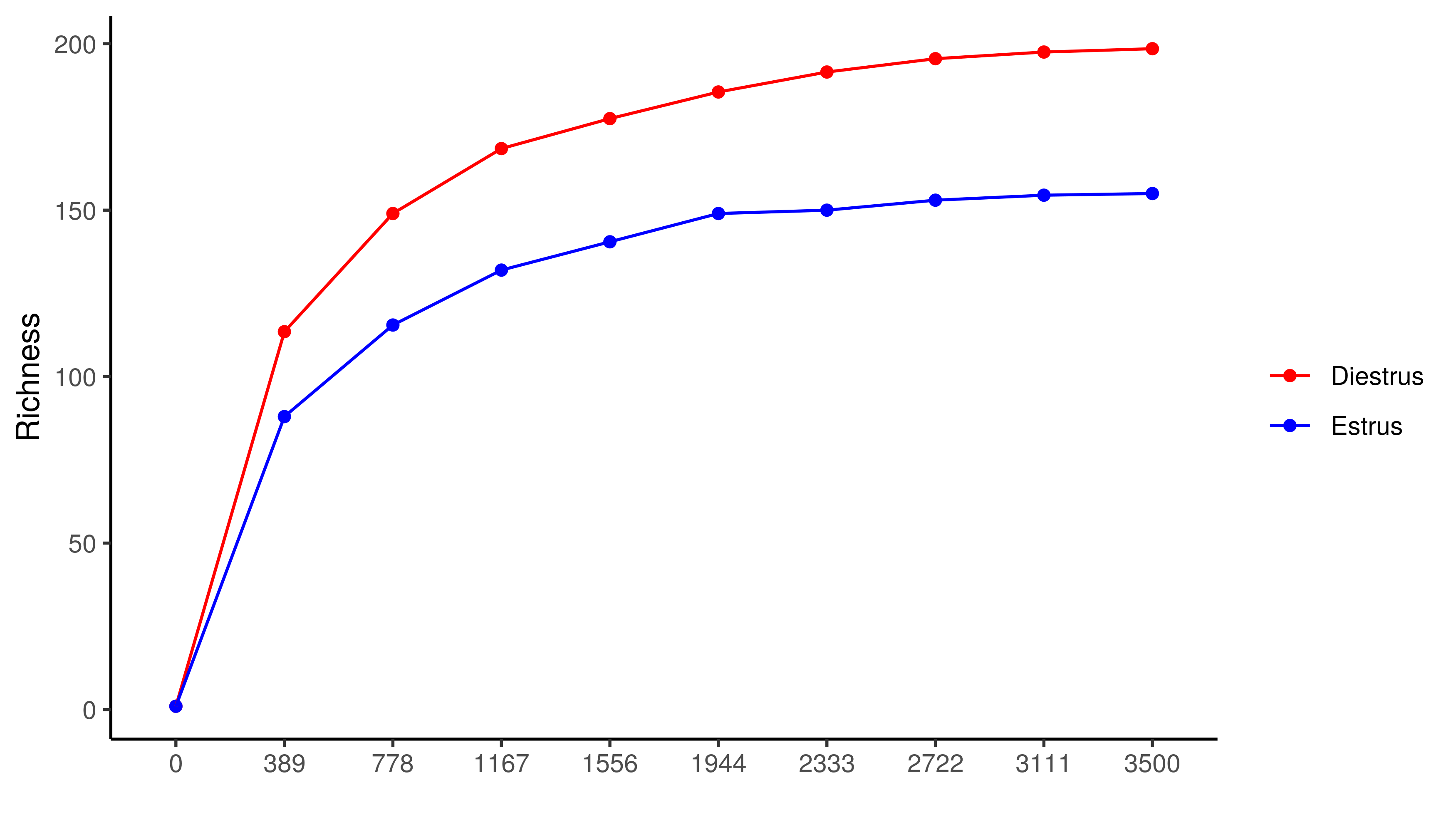

Supplement: Supplementary file 1 [file animals-10-02020-s001.zip › Supplementary Figure 1.tiff]

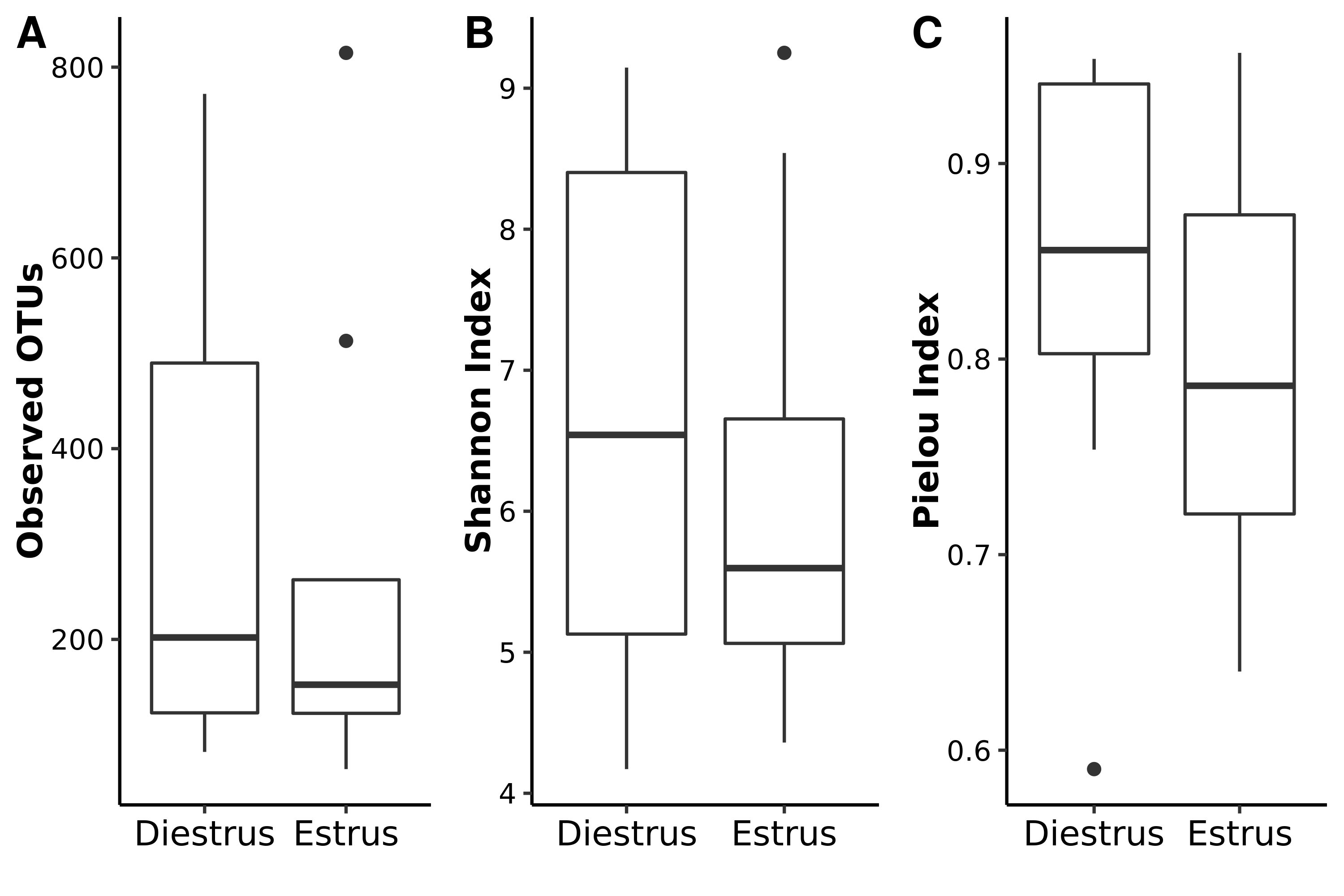

Supplement: Supplementary file 1 [file animals-10-02020-s001.zip › Supplementary Figure 2.tiff]

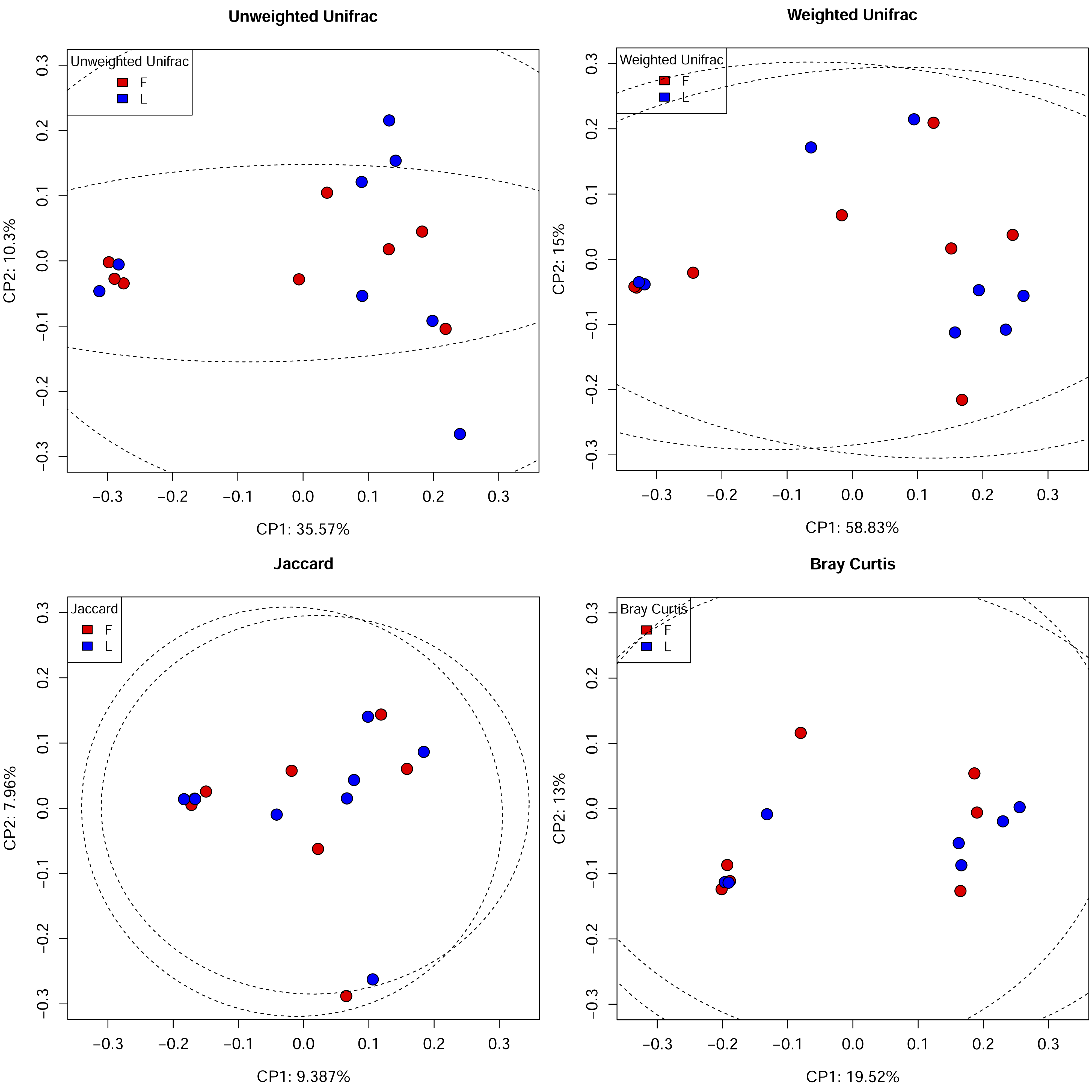

Supplement: Supplementary file 1 [file animals-10-02020-s001.zip › Supplementary Figure 3.tif]
